# Neutropthil-to-Lymphocyte Ratio as a Predictor of Postsurgical Intraabdominal Abscess in Children Operated for Acute Appendicitis

**DOI:** 10.3389/fped.2019.00424

**Published:** 2019-10-29

**Authors:** Carlos Delgado-Miguel, Antonio J. Muñoz-Serrano, Vanesa Núñez, Karla Estefanía, María Velayos, Miriam Miguel-Ferrero, Saturnino Barrena, Leopoldo Martínez

**Affiliations:** ^1^Department of Pediatric Surgery, University Hospital La Paz, Madrid, Spain; ^2^Institute for Biomedical Resarch La Paz (IdiPaz), Network for Maternal and Children Health (SAMID), Children's Hospital La Paz, Madrid, Spain

**Keywords:** neutrophil-to-lymphocyte ratio (NLR), acute appendicitis, abdominal abscess, children, appendectomy

## Abstract

**Aim of the study:** Postoperative intra-abdominal abscess (PIAA) is a frequent and severe complication of acute appendicitis (AA) with peritonitis. The neutrophil-to-lymphocyte ratio (NLR) is an inflammatory marker that has been related to the development of peritonitis; however, its diagnostic role in predicting PIAA has not been evaluated. This is the first study that analyzes the usefulness of NLR as a predictor of PIAA in children operated for AA.

**Material and Methods:** Retrospective observational study in children operated for AA in our institution during 2017–2018. Patients aged under 5 years or with incomplete laboratory determinations at hospital admission (blood count, C-reactive protein, and fibrinogen) were excluded. Demographic and laboratory parameters and the development of PIAA were analyzed. NLR was calculated by dividing the absolute number of neutrophils by the absolute number of lymphocytes. By means of ROC curves, we determined the sensitivity and specificity of the different laboratory parameters to predict the development of PIAA.

**Results:** A total of 388 patients aged 10.5 ± 2.9 year were included. Twenty (5.2%) developed PIAA. NLR presented an area under the curve (AUC) of 0.85, significantly higher than the determination of leukocytes (AUC 0.69, *p* < 0.001), neutrophils (AUC 0.74, *p* < 0.001), fibrinogen (AUC 0.68, *p* < 0.001) and C-reactive protein (AUC 0.73, *p* < 0.001). We estimated the optimal cut-off point of NLR > 10.5, with a sensitivity of 85% and a specificity of 75.2%.

**Conclusions:** NLR is the laboratory parameter with the highest sensitivity and specificity for predicting the development of PIAA in children operated for AA. It can be useful as a predictor of worse postoperative course.

## Introduction

Acute appendicitis (AA) is the most frequent surgical emergency in all age groups (1), with an estimated annual incidence of 100 per 100,000 inhabitants, with an overall risk of 8.6% for men and 6.7% for women (2). Its diagnosis depends to a great extent on the clinical assessment of the surgeon, although in the pediatric age it is often difficult to make an early diagnosis, due to the difficulties of doctor-patient communication and the absence of classic symptoms (3). Acute appendicitis requires immediate diagnosis and treatment, as any delay may lead to complicated appendicitis (gangrenous or perforated), with or without localized collection (4). Patients who undergo appendectomy for complicated appendicitis are more likely to develop post-operative complications, such as wound infection or intra-abdominal abscess (PIAA). It is estimated that PIAA can complicate up to 4.2% of non-perforated acute appendicitis (5) and between 6.7 to 28% of perforated appendicitis (6). PIAAs entail a significant increase in morbidity and mortality (7, 8) and are responsible for longer hospitalizations and/or re-admissions (9).

The role of different laboratory parameters (such as leukocyte count, absolute and relative number of neutrophils, and C-reactive protein) as potential predictors of PIAA has been studied, without conclusive results. On the other hand, decreased levels of plasma sodium on admission have been correlated with an increased risk of developing PIAA (10).

Recently, the neutrophil-to-lymphocyte ratio (NLR) has been postulated as an inflammatory marker in several abdominal pathologies, such as inflammatory bowel disease (11), colorectal cancer (12), and sepsis of abdominal origin (13). Likewise, its role as a predictor of complicated appendicitis in adult patients has been studied, with unconclusive results (14, 15). However, there are scarce studies that analyze the usefulness of NLR in pediatric patients (16).

The aim of this study was to examine the role of NLR in predicting the development of PIAA in children operated for AA.

## Materials and Methods

A retrospective observational study was performed in patients undergoing surgery for AA in our center between January 2017 and December 2018. Demographic variables (age and sex), laboratory variables and the development of PIAA were analyzed. Laboratory variables were obtained from the blood tests performed in the emergency department at the patient's arrival (before surgery), which included blood count (leukocytes, absolute and relative values of neutrophils, lymphocytes, monocytes, basophils and eosinophils), biochemistry (ionogram, glucose, urea, fibrinogen), and C-reactive protein. NLR was calculated by dividing the absolute number of neutrophils by the absolute number of lymphocytes. The development of intra-abdominal abscess was defined according to the criteria of the Centers for Disease Control and Prevention (CDCs) of the USA as “the presence of purulent material inside the abdominal cavity” (17). Patients were divided into two study groups according to the development or not of PIAA during the first postoperative month.

We included all patients between 5 and 16 years of age with an intraoperative diagnosis of AA. Patients aged under 5 years were excluded, due to the important physiological differences in the leukocyte count up to that age. Other exclusion criteria were incomplete laboratory tests at hospital admission and the absence of acute appendicitis when the surgery was performed.

In our center, we perform surgical treatment of all AA, with preoperative antibiotic therapy with Amoxicillin-Clavulanic acid. Post-operative antibiotic is only continued in gangrenous appendicitis (Amoxicillin-clavulanic acid for 5 days), and in appendicitis with peritonitis (Gentamicin, Metronidazole and Amoxicillin-Clavulanic acid for 7 days).

Ethical approval was not required due to the retrospective nature of this study, the absence of human or animal samples and the anonymous collection of analytical data, in line with institutional guidelines.

For the statistical analysis, the continuous variables were expressed as mean and standard deviation. To check whether the variables were normally distributed, the Kolmogorov-Smirnoff and Shapiro-Wilk tests were used. For the continuous variables normally distributed, the Student *t*-test of independent samples was used, and to analyze the continuous data not normally distributed, the Mann-Whitney test was used. The discrete variables were expressed as frequency and percentage, and were analyzed by the Chi square test, or Fisher's test when the first one could not be applied. Odds ratios (OR) were calculated with 95% confidence intervals. All statistical calculations were performed with two tails and the statistical significance was established with a value of *p* < 0.05. The sensitivity and specificity for the diagnosis of PIAA of the different collected laboratory parameters was determined by ROC curves. Subsequently, the cut point of maximum diagnostic accuracy for each analytical parameter was calculated using the Youden Index. The data was collected in Microsoft Excel software version 2010 (Redmond, WA, EE.UU.), and analyzed with SPSS Statistic version 22 (Chicago, IL, USA).

## Results

A total of 388 patients aged 10.5 ± 2.9 year were included. Twenty (5.2%) developed PIAA during the first postoperative month. There were no differences in age and sex between both groups, as shown in Table 1.

**Table 1:** Demographic characteristics.

	**Global (*n* = 386)**	**AA without IAA (*n* = 366)**	**AA with IAA (*n* = 20)**	***p***	**OR**
Age	10.4 ± 2.85	10.4 ± 2.88	9.80 ± 2.44	0.160	–
Sex
• Men	233 (60.4%)	220 (60.1%)	13 (65%)	0.427	1.23 (0.48–3.16)
• Women	153 (39.6%)	146 (39.9%)	7 (35%)		

Regarding hemogram parameters, high values of leukocytes, neutrophils, percentage of neutrophils and eosinophils were observed in the group of patients who presented PIAA, that were significantly higher than in patients without PIAA. On the contrary, this group presented higher values of lymphocytes and basophils, without statistically significant differences in monocytes and platelet numbers. NLR was significantly higher in patients with PIAA (15.6 ± 5.5 vs. 8.3 ± 5.8; *p* < 0.001). C-reactive protein levels were also higher in these patients (81.8 ± 72.8 vs. 33.1 ± 43.3, *p* < 0.001). With regards to the biochemical variables studied, plasma fibrinogen levels were higher in patients who developed PIAA (760.1 ± 208.5 vs. 446.1 ± 198.8; *p* = 0.015). No significant differences were observed in blood glucose levels or urea values between both groups of patients. When we analyzed electrolyte variables, we observed that the patients with PIAA showed significantly lower plasmatic sodium levels, without differences in the plasma levels of potassium and chlorine. All laboratory variables studied are shown in Table 2.

**Table 2:** Analytical parameters studied.

	**AA without IAA (*n* = 366)**	**AA with IAA (*n* = 20)**	***p***
Leukocytes	15.275 ± 4983	18.450 ± 4020	<0.001
Neutrophils (%)	12.247 ± 4829 78.2 ± 10.6	16.168 ± 3816 87.4 ± 5.1	<0.001 <0.001
Lymphocytes (%)	1.869 ± 828 13.8 ± 8.5	1,140 ± 419 6,3 ± 2,2	<0.001 <0.001
Monocytes (%)	940 ± 428 6.4 ± 2.3	1,143 ± 559 6.3 ± 3.1	0.125 0.866
Eosinófilos (%)	149 ± 222 1.1 ± 1.5	173 ± 236 0.3 ± 0.3	<0.001 <0.001
Basophils (%)	37 ± 29 0.3 ± 0.2	23 ± 18 0.1 ± 0.1	<0.001 0.003
Platelets	269.697 ± 71.480	281.200 ± 58.001	0.403
N-L Index	8.3 ± 5.8	15.6 ± 5.5	<0.001
Glucose	109.7 ± 17.5	96.1 ± 16.8	0.115
Urea	29.9 ± 10.9	27.8 ± 7.5	0.440
Fibrinogen	446.1 ± 198.8	760.1 ± 208.5	0.015
C-reactive protein	33.1 ± 43.3	81.8 ± 72.8	<0.001
Na	137.3 ± 4.0	135.3 ± 3.0	0.012
K	4.0 ± 0.4	4.5 ± 2.4	0.384
Cl	103.6 ± 6.1	102.6 ± 4.5	0.348

**Figure 1:**
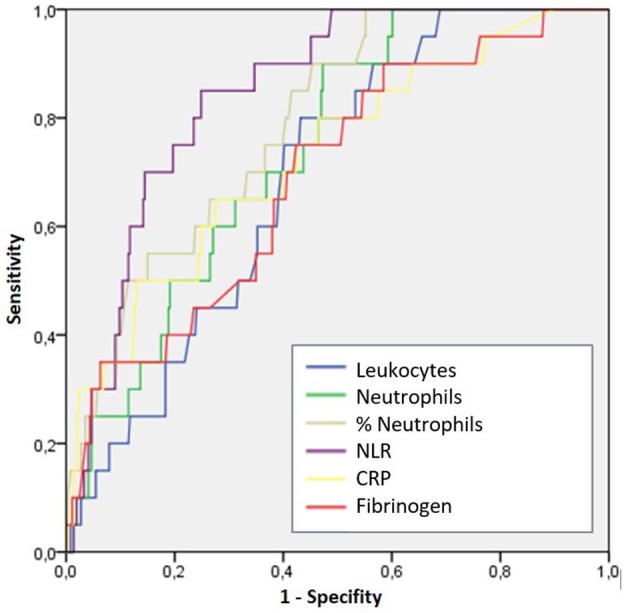
ROC curve for the diagnosis of PIAA.

When performing the sensitivity and specificity analysis using ROC curves for the diagnosis of PIAA, it was observed that NLR presented an area under the curve (AUC) of 0.85, significantly higher than the AUC of the absolute number of leukocytes (AUC 0.69, *p* < 0.001), neutrophils (AUC) 0.74, *p* < 0.001), fibrinogen (AUC 0.68, *p* < 0.001) and C-reactive protein (AUC 0.73, *p* < 0.001). Figure 1 shows the ROC curve for the diagnosis of PIAA. It was estimated the cut-off point of NLR >10.5, with a sensitivity and specificity of 85.0 and 75.2%, respectively. Both the absolute and relative value of neutrophils showed a sensitivity of 90%, but a specificity (53 and 54.8%, respectively) significantly lower than those of NLR. C-reactive protein presented a low sensitivity for the diagnosis of PIAA (65%), with a specificity of 72%, without statistically significant differences with those of NLR (*p* = 0.065). Table 3 shows the AUC of the parameters analyzed for the diagnosis of PIAA, as well as the cut points obtained by the Youden index and the sensitivity and specificity determined for each of them.

**Table 3:** Area under the ROC curve (AUC) for the diagnosis of intra-abdominal abcess.

	**AUC**	**Cut point**	**CI 95%**	**Sensitivity (%)**	**Specificity (%)**
Leukocytes	0.69	16.100	12.800–16.900	80.2	57.2
Neutrophils	0.74	12.698	11.0214–20.024	90.1	53.1
Neutrophils %	0.79	81.7	79.0–88.4	90.0	54.8
N-L Index	0.85	10.5	6.9–13.0	85.1	75.2
Platelets	0.56	250.000	181.000–294.000	75.2	39.5
C-reactive protein	0.73	35.6	9.1–78.1	65.0	71.9
Fibrinogen	0.68	452	391–660	75.1	57.2

## Discussion

This is the first study that analyzes the usefulness of NLR as a predictor of PIAA in children operated for AA.

The incidence of intra-abdominal abscesses of the sample (5.2%) is within what was expected, and it is in accordance with the literature data (5, 6).

Although AA is the most common surgical condition amongst children, diagnosis can be difficult sometimes due to the high prevalence of atypical symptoms and presentations. Therefore, it is important to find objective parameters that can help us to diagnose the disease and to prevent possible complications (18). Serradilla et al. studied retrospectively the risk factors for the development of PIAA in acute gangrenous appendicitis in children, determining as independent predictors hyponatremia at the time of diagnosis, perforation of the appendix and infection of the surgical wound, without finding conclusive results for other laboratory parameters studied (10).

The involvement of NLR as a diagnostic tool in AA has been previously studied in the adult population. Ishizuka et al. analyzed the relationship between NLR and the presence of gangrenous and perforated appendicitis in 314 adult patients, establishing a cut-off point> 8, with a sensitivity and specificity of 73 and 39%, respectively, which were lower than those obtained in our study (19).

At the time of our research, we only found one study that analyzes the usefulness of NLR in pediatric patients (16). Yazici et al. compared laboratory parameters in children with AA vs. children with non-specific abdominal pain. They determined a NLR cut-off point of 3.5 for the diagnosis of AA with a sensitivity of 90% and a specificity of 88%. However, the presence of peritonitis or the development of subsequent postoperative complications were not analyzed.

Our study has demonstrated the involvement of NLR as a predictor of PIAA in pediatric patients operated for acute appendicitis, with a sensitivity and a specificity superior to those of the laboratory parameters routinely studied, such as leukocytosis, neutrophilia and C-reactive protein. The pediatric surgeon should know its relevance as a tool to help in early diagnosis and treatment of PIAA, in order to decrease the high morbidity and mortality it generates. Due to its high availability in almost all pediatric emergency departments, it should be considered a fundamental diagnostic tool. In children with a high clinical suspicion of PIAA, with symptoms such as diarrhea (8), fever and non-tolerating a regular diet (20), high values of NLR prior to surgery (as determined by our study) should encourage the performance of an imaging test as soon as possible.

We are aware that this study presents several limitations, such as being an unicenter study and its retrospective design. Although the sample size allowed us to detect significant differences, studies with a greater number of patients are recommended in order to achieve higher statistically power. Finally, due to the physiological differences in the values of hemogram parameters in patients under 5 years of age, the results cannot be extrapolated to this population (21).

Our results should inspire new prospective studies, in order to develop strategies that could prevent the development of PIAA. For example, by adjusting the presurgical antibiotic protocol in patients with high risk of developing a PIAA. Children diagnosed of AA and with high levels of NLR on admission, could benefit from initiating a pre-surgical antibiotic treatment of a wider spectrum than those patients with low NLR values. A prospective study in children with AA, comparing different antibiotics protocols according to the risk of developing PIAA, based on presurgical NLR values, is about to be started in our center.

## Conclusions

NLR is the laboratory parameter with the highest sensitivity and specificity for predicting the development of PIAA in children operated for AA. It can be useful as a predictor of worse postoperative course.

## Data Availability Statement

All datasets generated for this study are included in the manuscript/supplementary files.

## Author Contributions

CD-M and AM-S contributed to the design, data collection, and statistical analysis of the study. KE and MV contributed to the data collection. VN, SB, and LM contributed in the analysis of the results. MM-F contributed to the revision of the manuscript.

### Conflict of Interest

The authors declare that the research was conducted in the absence of any commercial or financial relationships that could be construed as a potential conflict of interest.
